# On Iodognosis

**Published:** 1851-05

**Authors:** 


*On Iodognosis.*—M. Dorvault has published a series of researches on the chemical therapeutical, and medical properties of iodine. To these, as embracing the entire knowledge of all the properties of that substance, he has given the name of iodognosis, *iodoynosie.*

We here submit an abstract of the medical portion of these researches, from the *Gazette Medicale de Paris:—*

Iodine, as a therapeutic agent, according to M. Dorvault, is unimportant; it is to its combination as *iodides* that its medical value is due. Even when introduced alone into the system, its therapeutic effects are to be attributed to its combination with the alkalies which exist in the fluids of the body. Under either circumstances the terms *iodic medication* express the same fact. Iodide of potassium is taken by M. Dorvault as the type of iodides.

*Physiological Action of Iodides.*—Iodides belong to that class of therapeutic agents to which M. Dorvault gives the name of *chemico-catalytic*, and form its most striking representative. This proposition is founded on the following facts: If the animal fluids (blood, lymph, semen, milk,) or their proteic elements (albumen, fibrin, casein,) be subjected to the action of a solution of iodide of potassium, it will be seen to prevent their coagulation and dissolve them. In producing this effect the salt remains unaltered; it acts, therefore, by virtue of what chemists have called the *catalytic* force. The same may be shown to have obtained when employed in certain pathological cases. The salt may be detected unaltered in the blood or urine, or other secretions.

These facts have been observed by many other investigators, and all have found practically that iodide of potassium promotes secretion, increases the functions of the mucous glands of the alimentary canal, and of the liver, kidneys, skin, pancreas, parotid, etc.

Iodide of potassium is rapidly eliminated from the animal fluids. Dr. Scharlan (of Stettin) found that a patient, to whom he gave 53 grammes daily, eliminated 51 grains by his urine. The five grains lost were accounted for by the elimination of this salt by the saliva, sweat, and tears. Dr. Kramer satisfied himself, from his experiments, that six days sufficed for the complete elimination of this salt after its exhibition during 50 days. The researches of Dr. Marchal, at Vâl de Grace, also prove the rapid passage of iodide of potassium by the urine.

Iodine introduced into the system has been separated by the action of alkalies on the blood.

*Special Action of Iodides.*—The accidental or consecutive action of iodides has often been mistaken for their primary or efficient action. Some physiologists have considered iodine as a stimulant, others as a contra-stimulant. M. Dorvault observes that neither view expresses the exact truth. He admits a certain degree of general constitutional excitement under its employment; also that, in severe pains of the bones and other tumors, the action of iodine is sedative, by allaying pain. But in both these cases the stimulating and the sedative action are the consequence, not the cause, of the beneficial therapeutic agency of the remedy.

A third opinion, that iodine is alterative, M. Dorvault regards as nearer the true explanation, but as insufficient in fact, as the medicinal influence of the iodides is often seen after the first dose, therein differing from alteratives. M. Dorvault admits, however, the alterative action of some substances in which iodine exists in minute quantities,—*e. g.*, sponge, cod-liver oil, etc.

M. Dorvault also considers the purely chemical theory of the action of iodine as incorrect; his own opinion being, that the medicinal virtue of the iodides consists in their power of dissolving or further liquefying the *humors* of animal bodies, of separating their constituent or proteic elements, and disposing these to the formation of new products, such as coagula, false membranes, and pathological concretions: that the iodine and the potassium united both concur in the production of this result, by a special and peculiar chemico-physiological power which iodides possess of liquefying the fibrine of the blood without destroying the globules; while potash, ammonia, and other substances, dissolve the blood in all its parts.

*Therapeutic Action of Iodine. The pathological states in which it is employed.*—Goitre, scrofula, syphilis, skin diseases, white swelling, caries of the vertebræ, tabes mensenterica, rickets, phthisis, leucorrhœa, amenorrhœa, and chlorosis, cancer, cachexies, dropsy, poisoning, tumors, rheumatism, various chronic diseases, hypertrophy.—These are the forms of disease in which, M. Dorvault observes, the administration of iodine is indicated.—*Ib.*

